# Canine Endogenous Oxytocin Responses to Dog-Walking and Affiliative Human–Dog Interactions

**DOI:** 10.3390/ani9020051

**Published:** 2019-02-08

**Authors:** Lauren Powell, Kate M. Edwards, Adrian Bauman, Adam J. Guastella, Bradley Drayton, Emmanuel Stamatakis, Paul McGreevy

**Affiliations:** 1 Charles Perkins Centre, Prevention Research Collaboration, Sydney School of Public Health, Faculty of Medicine and Health, University of Sydney, Sydney, NSW 2050, Australia; adrian.bauman@sydney.edu.au (A.B.); bradley.drayton@sydney.edu.au (B.D.); emmanuel.stamatakis@sydney.edu.au (E.S.); 2 Charles Perkins Centre, Faculty of Health Sciences, University of Sydney, Sydney, NSW 2050, Australia; kate.edwards@sydney.edu.au; 3 Autism Clinic for Translational Research, Brain and Mind Centre, Central Clinical School, Sydney Medical School, University of Sydney, Camperdown, NSW 2006, Australia; adam.guastella@sydney.edu.au; 4 Sydney School of Veterinary Science, Faculty of Science, University of Sydney, Camperdown, NSW 2006, Australia; paul.mcgreevy@sydney.edu.au

**Keywords:** oxytocin, dog-walking, human–animal interaction, attachment, canine, social behavior

## Abstract

**Simple Summary:**

It is widely recognized that humans and dogs share a unique relationship. However, the biological mechanisms that may contribute to this bond between owners and their pet dogs are still unclear. As such, we measured the concentration of oxytocin, a hormone that is important in social bonding, in dogs before and after two different activities: dog-walking and human–dog interactions. We also investigated whether the strength of an owner’s attachment to their dog affected the dog’s oxytocin concentration. Contradicting our suppositions, the experiment showed that the concentration of dog oxytocin was not substantially different following either dog-walking or human–dog interactions. Additionally, the strength of the human–dog bond did not affect oxytocin concentrations. We suggest that more research is needed to fully understand the role of oxytocin in human–dog bonding.

**Abstract:**

Several studies suggest human–dog interactions elicit a positive effect on canine oxytocin concentrations. However, empirical investigations are scant and the joint influence of human–dog interaction and physical activity remains unexplored. The aims of the current study were to (a) examine the canine endogenous oxytocin response to owner-led dog-walking and affiliative human–dog interactions and (b) investigate the moderating effect of the owner-reported strength of the human–dog bond on such responses. Twenty-six dogs took part in a random order cross-over trial, involving dog-walking and human–dog interactions. Urinary samples were collected before and after each condition. The data were analyzed using linear mixed models with condition, order of conditions, condition duration, and latency from initiation of condition to urine sample collection considered as fixed effects, and the participant was considered a random effect. Canine urinary oxytocin concentrations did not differ significantly following dog-walking (mean change: −14.66 pg/mg Cr; 95% CI: −47.22, 17.90) or affiliative human–dog interactions (mean change: 6.94 pg/mg Cr; 95% CI: −26.99, 40.87). The reported strength of the human–dog bond did not significantly moderate the canine oxytocin response to either experimental condition. Contrary to our hypothesis, we did not observe evidence for a positive oxytocin response to dog-walking or human–dog interactions.

## 1. Introduction

Over recent years, scientific recognition of the complex social skills possessed by dogs has increased, with canine social cognition often being considered analogous to that of humans [1]. A prime example lies in the type of relationship between humans and dogs where, similar to a human infant, dogs often display behaviors indicative of attachment bonds, such as proximity-seeking during stress [2]. Although the biological mechanisms underlying dogs’ human-like social competence remain unclear, oxytocin is regularly proposed as a primary mediator [3].

Oxytocin is a hormone and neuropeptide produced centrally, in the hypothalamus, and peripherally, in organs such as the heart [4]. Evidence suggests that the release of central oxytocin inhibits the activation of neural stress pathways, such as the hypothalamic-pituitary adrenal (HPA) axis, and threat-processing centers, such as the amygdala [5]. Peripheral oxytocin release is also believed to inhibit stress reactivity, observed in the cardiovascular system via reduced heart rate and increased heart rate variability [6]. Oxytocin also plays an essential role in mediating the neural reward of social information [7]. These mechanisms are likely to explain, at least in part, the increase in canine pro-social behaviors observed with elevated oxytocin concentrations, including increased gaze towards the owner [8] and increased affiliative behaviors such as sniffing, licking, body contact, and play [9].

Much of the research investigating oxytocin responses and canine social behavior has used either exogenous oxytocin administration, to increase oxytocin concentrations, or a genetic approach, examining associations between genetic polymorphisms in the oxytocin receptor gene and canine behavior [10]. Moreover, several correlational studies have investigated the influence of human interaction on canine endogenous oxytocin, documenting an almost universal increase in oxytocin concentrations [11,12,13,14,15,16]. The literature is consistent in that only one study, to our knowledge, has reported no change in oxytocin following human interaction [9]. The interaction protocols of each study vary significantly, examining interaction types such as gaze [14], stroking, petting, talking [11,12], and free-form interaction in which the dog leads the interaction type [16]. One study also examined the influence of exercise on endogenous oxytocin concentrations in nine dogs and reported a positive effect after 15 min of “trotting” [13]. However, the combined effect of exercise and human–dog interaction through activities such as owner-led dog-walking remains undocumented.

Dogs’ behavior toward humans varies significantly with familiarity [17] and closeness of the relationship. For example, dogs may direct most of their attention toward a familiar person who is primarily responsible for their care (e.g., in the form of exercising and feeding) compared to a familiar person who is responsible for less than half their care [18]. Given these behavioral differences, it is plausible the strength of attachment between owner and dog may also influence a dog’s oxytocin response to human interaction. Pilot data suggest a positive correlation exists between owner-reported strength of the human–dog bond and canine oxytocin concentrations. Alternatively, the owner’s perception of the difficulty in caring for their dog or the likelihood of their dog making a mess was negatively correlated with canine oxytocin concentrations [19].

Only a limited number of empirical investigations have examined endogenous oxytocin concentrations following human–dog interactions, and the sample size used in each study are typically small [9,11,13,14]. The primary aim of the current study was to investigate the canine endogenous oxytocin response to owner-led dog-walking and affiliative human–dog interaction. A secondary aim was to investigate the moderating effect of the owner-reported strength of the human–dog bond on canine oxytocin responses.

## 2. Materials and Methods

### 2.1. Participants

From June 2017 to May 2018 in Sydney, Australia, 30 owners and their dogs were recruited through social media and newspaper advertisements, word-of-mouth, and printed materials distributed in dog-friendly parks and veterinary clinics. Dogs were eligible to participate if they were aged one year or older; not in the last quintile of expected lifespan for their breed; the only dog in the household, to avoid the possible influence of conspecific interaction; and male, to aid sample collection. The dog also had to be walked regularly (for a minimum of 15 min, four times per week). Ethical approval was obtained from the University of Sydney Human Research Ethics Committee (2017/215) and Animal Ethics Committee (2017/1161). The study was registered with the Australian New Zealand Clinical Trials Registry (ACTRN12617000966392). Informed written consent was provided by the owner on behalf of themselves and their dog.

Twenty-nine of the 30 recruited dogs completed both conditions, with one dog excluded due to owner nonresponse. Most dogs (*n* = 24, 92%) were neutered, and the mean age was 6.5 years (SD 3.34). The dogs’ breeds are provided in the Supplementary Materials (Table S1). The majority of owners were female (*n* = 24, male: *n* = 5) with a mean age of 47.0 years (SD 15.2).

### 2.2. Human–Dog Bond Questionnaire

Owners were required to complete the Monash Dog Owners Relationship Scale (MDORS) [20]. The MDORS questionnaire quantifies the owner-reported strength of the human–dog relationship by investigating the owner’s perceived time spent in shared activities, attachment between human and dog, and costs associated with dog ownership. The question “How often do you take your dog in the car?” was excluded as not all owners in the cohort had access to a car [19]. A total MDORS score was then calculated by reserve scoring items in the Dog–Owner Interaction and Perceived Emotional Closeness subscales and then averaging the total score of all items. In one instance where a participant did not answer a question, we extracted the age (10-year band) mean score for the relevant sex and assigned this value [21].

### 2.3. Experimental Conditions

This study forms part of a larger, random order cross-over trial, with the human-centered outcomes reported elsewhere (publication under review). In the present manuscript, canine oxytocin concentrations were investigated following two experimental conditions: dog-walking (DW) and affiliative human–dog interaction (H-DI). Randomization.com, an online program, was used to randomly pre-determine the sequence of conditions [22] using random permutations with no blocking restrictions. Fourteen participants completed DW first while the remaining 15 participants completed H-DI first.

Owners were instructed not to feed their dog and to avoid interactions with him, including both verbal and physical communication, in the two hours prior to each experimental condition. Both conditions were performed at the same time of day in the dog’s home or surrounding area, in the case of DW. Upon arrival at the participant’s home, the researcher avoided interaction with the dog as much as possible. A minimum 24 h washout period was required between conditions.

#### 2.3.1. Dog-Walking (DW)

Prior to initiating the condition, owners were instructed to avoid interactions with other people or dogs during the walk. Owners then walked their dog on-leash, at their habitual pace, on a route familiar to both human and dog for approximately 15 min. A researcher recorded the exact distance walked (km) and time (min) using the Strava smartphone app [23]. The researcher ensured that the dog did not interact with other humans or dogs during this walk.

#### 2.3.2. Affiliative Human–Dog Interaction (H-DI)

Owners interacted with their dog for a 15 min period in their home or backyard. The type of interaction was not specified and could include mechanisms, such as patting, talking to, and gazing. If the dog initiated a more active interaction style, such as low-key play, the owners were told that they could engage with him. However, they were instructed to avoid games such as “fetch” that would increase the activity levels of the owner. Most owners used a combination of interaction techniques, with physical contact, verbal communication, and play being the most common.

### 2.4. Hormone Analysis

#### 2.4.1. Urinary Oxytocin

Urine samples were collected from the dog immediately before and approximately 30 min after the start of each condition, based on previous research that suggests urinary oxytocin should be measured 30–90 min after exposure to a stimuli [13]. Samples were collected by holding a plastic tray underneath the dog while they urinated spontaneously. The samples were then kept on ice during transportation back to the laboratory before storage at −80 °C until analysis. Urine was successfully collected from 27 dogs, between 27:09 and 43:36 after initiating the condition (mean = 32 min), with the remaining two dogs excluded from analyses. Three dogs did not provide samples after one condition, resulting in missing data for this condition.

Urinary oxytocin concentration was measured using a commercial OT-ELISA kit (ENZO, NY, USA). Previous research has found this kit to be valid for measuring canine urinary oxytocin. Extracted urine and raw urine produced comparable results, although the latter required dilution prior to analysis. Therefore, to ensure the concentration of oxytocin fell within the kit’s readable range, urine was diluted either 5 or 8 times. The rate of dilution varied depending on the concentration of urine (Dr Takefumi Kikusui, Azabu University, Sagamihara, Japan, personal communication). All measurements were performed in duplicate, with the mean oxytocin concentration recorded. In the case that the duplicates produced substantially different results, two further duplicates were analyzed and the mean value recorded. Samples with a concentration greater than 3 standard deviations from the relevant pre or post sample group mean were removed, resulting in the exclusion of one dog. Additionally, one sample did not return a valid reading, which resulted in one dog displaying missing data for one condition.

#### 2.4.2. Urinary Creatinine

Urinary creatinine concentration was determined using the Beckman Coulter modification of the Jaffe reaction [24]. All measurements were performed in duplicates. Urinary oxytocin concentrations were then standardized to the creatinine concentration to control for variation in urine concentration [25]. Data are expressed here as the oxytocin to creatinine ratio (pg/mg Cr) [14].

### 2.5. Statistical Methods

Change in urinary oxytocin was calculated by subtracting the pre-condition concentration from the post-condition value. Linear mixed models were used to examine the effect of each condition on urinary oxytocin concentrations. The condition, period (order of conditions), condition duration (min), and latency from initiation of condition to urine sample collection (min) were considered as fixed effects, and the participant was considered a random effect. 

Sub-group analyses examining the influence of dog age, breed, and MDORS scores on endogenous oxytocin responses were decided and published a priori [26]. Dog breeds were grouped into three categories: sporting and herding, terrier, and toy, based on the American Kennel Club [27] breed groupings. The “sporting and herding” category includes breed listed under the “sporting” or “herding” groups. For dog age and MDORS score, participants were divided in two groups, using the group medians as classification cut-offs. This study was not statistically powered to investigate the effect of owner sex on canine oxytocin concentrations. Statistical analysis was conducted in SPSS version 24 (IBM, Armonk, NY, USA), and *p* < 0.05 was considered statistically significant.

## 3. Results

### 3.1. Differences in Urinary Oxytocin

**Figure 1:**
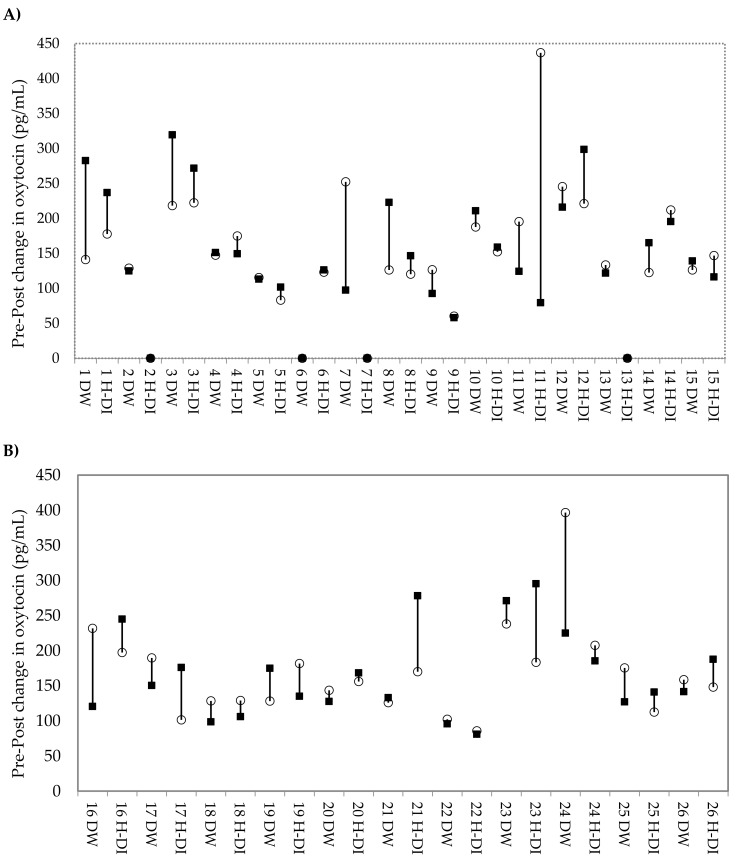
Change in urinary oxytocin concentration from pre to post condition for each dog. DW: dog-walking; H-DI: human–dog interaction. ○ indicates the pre-condition concentration, and ▪ indicates the post-condition concentration. In the case ○ and ▪, both fall on 0, which indicates missing data (*n* = 4 conditions). Dogs are displayed in order of ascending total MDORS score, from smallest to largest. (**A**) Dogs whose owners reported below median levels of attachment. (**B**) Dogs whose owners are reported above the median levels of attachment.

Changes in urinary oxytocin concentrations were not significantly different following dog-walking (DW) (estimated mean change: −14.66 pg/mg Cr; 95% CI: −47.22, 17.90) or affiliative human–dog interaction (H-DI) (estimated mean change: 6.94 pg/mg Cr; 95% CI: −26.99, 40.89). Pairwise comparisons reveal the mean difference in pre–post changes of oxytocin between DW and H-DI was also non-significant. The period (order of conditions), duration of condition (min), and latency from initiation of stimulus to urine sample collection (min) did not significantly affect the oxytocin response. Confirming this, Figure 1 displays the change in oxytocin concentration from pre–post condition for each individual dog, with no clear pattern apparent.

### 3.2. MDORS

**Figure 2:**
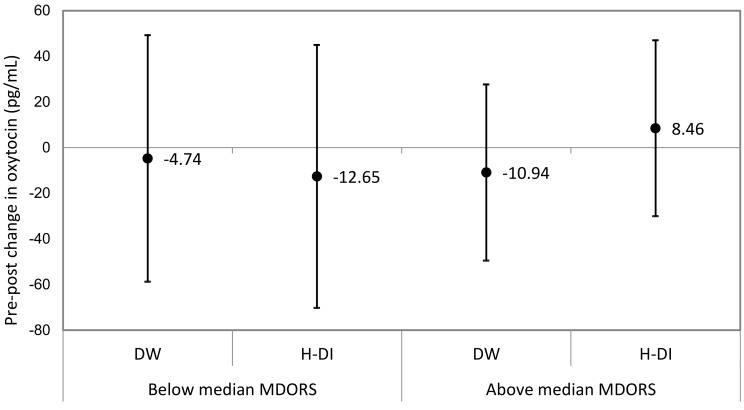
Sub-group analyses of estimated mean pre–post change of urinary oxytocin concentration by the strength of the human–dog bond. DW: dog-walking; H-DI: human–dog interaction.

The total MDORS score (mean: 85.6%, SD: 6.56%) did not moderate the canine oxytocin response to human–dog interactions. In both dogs whose owners reported above median levels of attachment and below median levels of attachment, there was no significant change in urinary oxytocin concentrations following DW and H-DI (Figure 2). 

### 3.3. Sensitivity Analysis

#### 3.3.1. Dog Breed Group

The change in urinary oxytocin concentrations were not significantly different following DW or H-DI in any of the three groups of dog breeds (Table S2).

#### 3.3.2. Dog Age

Dog age did not significantly affect the urinary oxytocin response to DW or H-DI. For example, following H-DI, both dogs aged below and above the median age displayed a non-significant increase of 6.79 pg/mg Cr (95% CI: −47.72, 61.30) and 27.56 pg/mg Cr (95% CI: −4.23, 59.35) respectively (Table S2).

## 4. Discussion

The primary aim of this study was to examine responses in the concentration of canine endogenous oxytocin to dog-walking and affiliative human–dog interactions. Contrary to most of the current literature [11,12,13,14,15,16], we did not observe a significant effect of owner-led walking or affiliative human–dog interactions on urinary oxytocin concentrations. Our results align with one previous study that reported no effect of human–dog interactions on urinary oxytocin concentrations when examining affiliation between owner and dog, or the proximity of dogs to their owners [9].

A secondary aim was to investigate the moderating effect of the owner-reported strength of the human–dog bond on canine oxytocin responses to dog-walking or affiliative human–dog interactions. Again, there was no significant change in oxytocin concentration following interactions in dogs whose owners reported greater or lower levels of attachment to their dogs. These findings are discordant with those of Handlin and Nilsson [19], who reported a positive association between canine oxytocin concentrations and the total MDORS score. A positive correlation has also been documented with individual indicators of the human–dog bond, such as the frequency of kissing the dog [19]. However, kissing dogs may be a feature of attachment that only certain owners have with their dogs and may not be representative of all owner–dog bonds. Furthermore, some dogs do not enjoy having humans blow or breathe on their faces [28], so they may self-select out of such face-to-face interactions.

The effects of oxytocin differ based on sex in both human and non-human animals [29]. Recent reports highlight a positive effect of oxytocin administration on urinary oxytocin concentrations in female dogs only, with males displaying no change in concentration following intranasal administration and a 30-min human–dog interaction [8]. Several studies have also documented the positive effects of oxytocin administration on human-directed gazing behavior in female dogs only [14,30]. Therefore, it is plausible that the present use of an entirely male sample may have influenced results, possibly explaining the null findings. Differences in the samples analyzed, namely, the use of urinary samples compared to the use of plasma samples in most previous studies [12,15,31] may also contribute to the discrepancies between our findings and those of previous studies. Alternatively, it has been suggested that replicating findings from human–animal interaction research is difficult due to small effect sizes, small sample sizes, and the variety of research designs seen throughout the literature [32]. It is also possible that selective reporting of findings based on their direction and statistical significance may have skewed the research on human–animal interactions [32]. Therefore, the relatively consistent positive influence of human interaction on canine endogenous oxytocin concentrations seen throughout the literature may, to some extent, be a reflection of publication bias, in which significant results are more likely to be published than null results [33].

To our knowledge, this is the first study to examine the influence of human–dog interactions on canine endogenous oxytocin concentrations within the dog’s home environment. It is plausible that human–dog interactions performed in clinical settings for research purposes are not indicative of authentic interactions between an owner and their companion dog. Therefore, testing privately owned pet dogs in the home environment, as in the current design, is likely to better simulate “real-world” conditions. The use of a within-subject, cross-over study design may also be considered a strength of the current study. 

However, a number of possible limitations must be noted. Firstly, differences in the mechanisms underlying central and peripheral oxytocin release remain unclear [5]. Currently, it is unknown how central and peripheral release patterns differ and whether peripheral oxytocin concentrations are indicative of central oxytocin concentrations [34,35]. Therefore, findings based on peripheral oxytocin concentrations should be interpreted with caution [34]. Secondly, it is possible that canine oxytocin responses were influenced by the researcher’s presence during experimental conditions, despite the study protocol having been designed to reduce any arousing effect of the researcher’s arrival at the home and despite the concerted efforts of researchers not to interact with the dog. For example, the researcher’s arrival at the participant’s home, approximately 15 min prior to baseline sample collection, could have elevated baseline concentrations, thereby reducing the observed effect of stimuli on oxytocin concentrations. The use of a convenience sampling method may have produced a sample of dog owners with greater levels of attachment to their dog compared to the general population. Therefore, it is not known whether our results based on a binary divide of participants into “low” and “high” levels of attachment can be extrapolated to the general population. A lack of detailed information regarding the dogs’ breeds, particularly in the case of cross-breeds, also limits the sub-division of dogs into breed groups because this relied on attempts to classify the dogs according to putative breed or cross. Similarly, there are limitations in using the group age median as a cut-off given the differences in life expectancy for various breeds. However, our study did not include any breeds with drastically shorter life expectancies, and we can assume that dogs aged below the median of 7 years were in the early or mid-stages of their life, while dogs aged over 7 years were likely to be in the mid to late stages of life. Finally, due to pressure on resources, urine was collected at the minimum time point of the suggested range (approximately 30 min). Additionally, interactions were not coded, meaning we were not able to examine possible associations between the oxytocin response and the type of interaction or time spent engaged in each interaction behavior.

Future research is warranted to replicate and extend our findings. To begin with, it may be helpful to apply the current methodology to female dogs. Studies investigating the moderating effect of individual differences on the canine oxytocin response would also be of interest, including the influence of genetics, early life experience, and the attachment style of the dog [36]. Accordingly, previous studies have highlighted an association between genetic variation in the oxytocin receptor gene and human-directed social behavior following intranasal oxytocin administration [37]. The need for such studies is further supported by recent reports that oxytocin does not elicit purely pro-social or anxiolytic effects on canine behavior. For example, Pekkin and Hänninen [38] documented a positive association between baseline concentrations of urinary oxytocin and general fearfulness, noise fear frequency, and reactivity.

To conclude, dog-walking and affiliative human–dog interactions did not have an effect on urinary oxytocin concentrations in this sample of male, privately owned, companion dogs. Further research is needed to improve and optimize oxytocin measurement standards and to establish the effect of different types of human–dog interactions on endogenous canine oxytocin concentrations in larger samples including both male and female dogs.

## Supplementary Materials

The following are available online at http://www.mdpi.com/2076-2615/9/2/51/s1. Table S1: Dog breeds (*n* = 26); Table S2: Sub-group analyses of estimated mean change of urinary oxytocin concentration from pre- to post-condition by dog breed and age.
