# Supplementary material for: Canine Endogenous Oxytocin Responses to Dog-Walking and Affiliative Human–Dog Interactions
Source: Animals (Basel). 2019 Feb 8;9(2):51. doi: 10.3390/ani9020051 (PMC6406532; doi:10.3390/ani9020051)
Supplement: Supplementary file 1 [file animals-09-00051-s001.pdf]

Supplementary Materials

Table S1. Dog breeds (n=26)

Dog breed	Frequency	Breed Group
Border Collie	1	Herding
Border Collie cross	2	Herding
English Pointer	1	Sporting
English Springer Spaniel	1	Sporting
Jack Russell	3	Terrier
Jack Russell cross	2	Terrier
Labrador	3	Sporting
Miniature Schnauzer	1	Terrier
Poodle	1	Toy
Toy poodle cross	2	Toy
Poodle cross Labrador/Golden Retriever	3	Sporting
Pug	1	Toy
Shih-Tzu cross	1	Toy
Staffordshire bull terrier	1	Terrier
Staffordshire bull terrier cross	3	Terrier

Table S2. Sub-group analyses of estimated mean change of urinary oxytocin concentration from pre to post-condition by dog breed and age

Condition (I)	Estimated mean change (pg/mg) (95% CI)
Breed	
Sporting and herding (n=11)	
DW	-23.11 (-81.19, 34.98)
H-DI	-8.33 (-67.02, 50.35)
Terrier (n=10)	
DW	-23.79 (-69.28, 21.70)
H-DI	18.14 (-33.41, 69.68)
Toy (n=5)	
DW	52.18 (-8.68, 113.03)
H-DI	23.92 (-36.94, 84.77)
Age	
Less than or equal to median age (7 years) (n=16)	
DW	-42.92 (-94.20, 8.35)

H-DI	6.79 (-47.72, 61.30)
Above median age (7 years) (n=10)	
DW	0.83 (-30.97, 32.62)
H-DI	27.56 (-4.23, 59.35)

DW: dog-walking, *H-DI:* human-dog interaction
n; number of participants in sample
Condition duration (min) and latency of urine collection evaluated at the mean value. Mean change for each condition was estimated using a linear mixed model with a) participant as a random effect; b) condition, order of conditions, condition duration (min) and latency of urine sample collection (min) as fixed effects
Adjustment for multiple comparisons: least significant difference.
